# Supplementary material for: Pre-Cambrian origin of envelope-carrying retrotransposons in metazoans
Source: eLife. 2026 Aug 28;14:RP108449. doi: 10.7554/eLife.108449 (PMC13524379; doi:10.7554/eLife.108449)
Supplement: Peer review history

# Editor report: eLife Assessment

**DOI:** 10.7554/eLife.108449.3.sa0

**Authors:** Detlef Weigel

**Affiliations:** Max Planck Institute for Biology Tübingen Germany

**Keywords:** Convincing, Important

This **important** study provides **convincing** evidence that envelope-carrying Ty3/gypsy retrotransposons (errantiviruses) are ancient and widespread across nearly all major animal phyla, with distribution in many lineages that are consistent with recent or ongoing genomic expansion. Using comprehensive phylogenetic and AlphaFold2-based structural analyses, together with new host-taxonomy concordance tests, the authors show that these elements independently acquired membrane fusion proteins early in metazoan evolution, likely predating the bilaterian-non-bilaterian split. The work offers significant insights into the deep evolutionary roots of retroelement-envelope associations and the origins of retroviruses.

# Referee report: Reviewer #1 (Public review)

**DOI:** 10.7554/eLife.108449.3.sa1

[Editors' note: this version has been assessed by the Reviewing Editor without further input from the original reviewers. The revision clarifies terminology, more carefully distinguishes element intactness from demonstrated transpositional activity, and better acknowledges the roles of lineage-specific loss and localized horizontal transfer alongside vertical inheritance.]

Summary:

This manuscript provides a comprehensive systematic analysis of envelope-containing Ty3/gypsy retrotransposons (errantiviruses) across metazoan genomes, including both invertebrates and ancient animal lineages. Using iterative tBLASTn mining of over 1,900 genomes, the authors catalog 1,512 intact retrotransposons with uninterrupted gag, pol, and env open reading frames. They show that these elements are widespread-present in most metazoan phyla, including cnidarians, ctenophores, and tunicates-with active proliferation indicated by their multicopy status. Phylogenetic analyses distinguish "ancient" and "insect" errantivirus clades, while structural characterization (including AlphaFold2 modeling) reveals two major env types: paramyxovirus F-like and herpesvirus gB-like proteins. Although bot envelope types were identified in previous analyses two decades ago, the evolutionary provenance of these envelope genes was almost rudimentary and anecdotal (I can say this because I authored one of these studies). The results in the present study support an ancient origin for env acquisition in metazoan Ty3/gypsy elements, with subsequent vertical inheritance and limited recombination between env and pol domains. The paper also proposes an expanded definition of 'errantivirus' for env-carrying Ty3/gypsy elements outside Drosophila.

Strengths:

(1) Comprehensive Genomic Survey:

The breadth of the genome search across non-model metazoan phyla yields an impressive dataset covering evolutionary breadth, with clear documentation of search iterations and validation criteria for intact elements.

(2) Robust Phylogenetic Inference:

The use of maximum likelihood trees on both pol and env domains, with thorough congruence analysis, convincingly separates ancient from lineage-specific elements and demonstrates co-evolution of env and pol within clades.

(3) Structural Insights:

AlphaFold2-based predictions provide high-confidence structural evidence that both env types have retained fusion-competent architectures, supporting the hypothesis of preserved functional potential.

(4) Novelty and Scope:

The study challenges previous assumptions of insect-centric or recent env acquisition and makes a compelling case for a Pre-Cambrian origin, significantly advancing our understanding of animal retroelement diversity and evolution. THIS IS A MAJOR ADVANCE.

(5) Data Transparency:

I appreciate that all data, code, and predicted structures are made openly available, facilitating reproducibility and future comparative analyses.

Original Major Weaknesses:

(1) Functional Evidence Gaps:

The work rests largely on sequence and structure prediction. No direct expression or experimental validation of envelope gene function or infectivity outside Drosophila is attempted, which would be valuable to corroborate the inferred roles of these glycoproteins in non-insect lineages. At least for some of these species, there are RNA-seq datasets that could be leveraged.

(2) Horizontal Transfer vs. Loss Hypotheses:

The discussion argues primarily for vertical inheritance, but the somewhat sporadic phylogenetic distributions and long-branch effects suggest that loss and possibly rare horizontal events may contribute more than acknowledged. Explicit quantitative tests for horizontal transfer, or reconciliation analyses, would strengthen this conclusion. It's also worth pointing out that, unlike retrotransposons that can be found in genomes, any potential related viral envelopes must, by definition, have a spottier distribution due to sampling. I don't think this challenges any of the conclusions, but it must be acknowledged as something that could affect the strength of this conclusion

(3) Limited Taxon Sampling for Certain Phyla:

Despite the impressive breadth, some ancient lineages (e.g., Porifera, Echinodermata) are negative, but the manuscript does not fully explore whether this reflects real biological absence, assembly quality, or insufficient sampling. A more systematic treatment of negative findings would clarify claims of ubiquity. However, I also believe this falls beyond the scope of this study.

(4) Mechanistic Ambiguity:

The proposed model that env-containing elements exploit ovarian somatic niches is plausible but extrapolated from Drosophila data; for most taxa, actual tissue specificity, lifecycle, or host interaction mechanisms remain speculative and, to me, a bit unreasonable.

# Referee report: Reviewer #2 (Public review)

**DOI:** 10.7554/eLife.108449.3.sa2

Summary:

The authors first surveyed metazoan genomes to identify homologs of Drosophila errantiviruses and classified them into two groups, "insect" and "ancient" elements, supporting the hypothesis of an early evolutionary origin for these retrotransposons. They subsequently identified two distinct types of envelope proteins, one resembling the glycoprotein F of paramyxoviruses and the other akin to the glycoprotein B of herpesviruses. Despite differences in their primary amino acid sequences, these proteins display notable structural similarity in their predicted domain architectures. The congruence between the phylogenies of the envelope and pol genes further supports the ancient origin of the envelope genes, challenging earlier hypotheses that proposed recent recombination events with baculoviruses. Additional analysis of the Pol "bridge region" corroborated the divergence among these elements, consistent with a pattern of limited cross-species recombination. Finally, by comparing these elements with non-envelope-containing Gypsy retrotransposons, the authors concluded that errantiviruses originated from multiple elements independently.

Strengths:

The conclusions of this study are based on a comprehensive collection of errantiviruses identified across a wide range of metazoan genomes. These findings are further supported by multiple lines of evidence, including phylogenetic congruence and the diverse evolutionary origins of envelope genes. AlphaFold2-assisted protein domain structure analyses also provided key insights into the characterization of these elements. Together, these results present a compelling case that errantiviruses arose independently through multiple evolutionary events, extending well beyond previous hypotheses.

Original Weaknesses:

It would be beneficial to emphasize in the Abstract the potential impact of this work by more clearly articulating the current knowledge gap in the field. While the second paragraph of the Introduction briefly touches on this point, highlighting the broader significance in the Abstract would better capture readers' interest. Additionally, some methodological choices would benefit from clearer justification and explanation. For instance, in Figure 6, the selection of the bridge region/RNase H domain is not explicitly explained, leaving the rationale for its choice unclear.

# Referee report: Reviewer #3 (Public review)

**DOI:** 10.7554/eLife.108449.3.sa3

Summary and Significance:

In this work, Cary and Hayashi address the important question of when, in evolution, certain mobile genetic elements (Ty3/gypsy-like non-LTR retrotransposons) associated with certain membrane fusion proteins (viral glycoprotein F or B-like proteins), which could allow these mobile genetic elements to be transferred between individual cells of a given host. It is debated in the literature whether the acquisition of membrane fusion proteins by non-LTR retrotransposons is a rather recent phenomenon that separately occurred in the ancestors of certain host species or whether the association with membrane fusion proteins is a much more ancient one, pre-dating the Cambrian explosion. Obviously, this question also touches upon the origin of the retroviruses, which can spread between individuals of a given host but seem restricted to vertebrates. Based on convincing data, Cary and Hayashi argue that an ancient association of non-LTR retrotransposons with membrane fusion proteins is most probable.

Strengths:

The authors take the smart approach to systematically retrieve apparently complete, intact, and recently functional Ty3/gypsy-like non-LTR retrotransposons that, next to their characteristic gag and pol genes, additionally carry sequences that are homologous to viral glycoprotein F (env-F) or viral glycoprotein B (env-B). They then construct and compare phylogenetic trees of the host species and individual encoded proteins and protein domains, where 3D-structure calculations and other features explain and corroborate the clustering within the phylogenetic trees. Congruence of phylogenetic trees and correlation of structural features is then taken as evidence for an infrequent recombination and a long-term co-evolution of the reverse transcriptase (encoded by the pol gene) and its respective putative membrane fusion gene (encoded by env-F or env-B). Importantly, the env-F and env-B containing retrotransposons do not form a monophyletic group among the Ty3/gypsy-like non-LTR retrotransposons, but are scattered throughout, supporting the idea of an originally ancient association followed by a random loss of env-F/env-B in individual branches of the tree (and rather rare re-associations via more recent recombinations).

# Author comment: Author response

**DOI:** 10.7554/eLife.108449.3.sa4

**Authors:** Rippei Hayashi, Shashank Chary

**Affiliations:** The John Curtin School of Medical Research, The Australian National University Canberra Australia; The John Curtin School of Medical Research, The Australian National University Canberra Australia

The following is the authors’ response to the original reviews.

In the revised manuscript, we have clarified several points that were raised by the reviewers. First, we now state more explicitly that the presence of intact *env*-containing *Ty3/gypsy* retrotransposons does not by itself demonstrate their mechanism of transmission, tissue specificity, infectivity, or current activity. We have therefore revised the wording throughout the manuscript to distinguish intact element structure and multicopy genomic expansion from experimentally demonstrated activity.

Second, we performed targeted host-taxonomy concordance analyses on selected clades of the POL RT tree. These analyses do not exclude local horizontal transfer, particularly between closely related hosts, but they show that horizontal transfer alone is insufficient to explain the broader host-taxonomic structure observed across the dataset.

Third, we incorporated representative viral and retroelement-associated fusogen proteins into our F-type ENV phylogenetic analysis and HSV/gB-type ENV structural comparison. These additions place the ENV proteins associated with *Ty3/gypsy* elements in a broader evolutionary context and strengthen the conclusion that these ENV associations are deeply diverged rather than recent derivatives of a single sampled viral lineage.

Fourth, we added two each of entirely new Supplementary figures (S5 and S7) and Tables (S2 and S3) and substantially modified now Supplementary figure S8. Other figures have also been modified only to increase readability. The four tables from the original manuscript have not been modified although their numbering has changed.

We believe that the revised manuscript is substantially improved in clarity, terminology and interpretive precision, while retaining the central conclusion that the association between *env*-like genes and *Ty3/gypsy* retrotransposons is ancient in metazoan evolution. Sincerely,

**Public Reviews:**

**Reviewer #1 (Public review):**

Summary:

This manuscript provides a comprehensive systematic analysis of envelope-containing Ty3/gypsy retrotransposons (errantiviruses) across metazoan genomes, including both invertebrates and ancient animal lineages. Using iterative tBLASTn mining of over 1,900 genomes, the authors catalog 1,512 intact retrotransposons with uninterrupted gag, pol, and env open reading frames. They show that these elements are widespread present in most metazoan phyla, including cnidarians, ctenophores, and tunicates-with active proliferation indicated by their multicopy status. Phylogenetic analyses distinguish "ancient" and "insect" errantivirus clades, while structural characterization (including AlphaFold2 modeling) reveals two major env types: paramyxovirus F-like and herpesvirus gB-like proteins. Although bot envelope types were identified in previous analyses two decades ago, the evolutionary provenance of these envelope genes was almost rudimentary and anecdotal (I can say this because I authored one of these studies). The results in the present study support an ancient origin for env acquisition in metazoan Ty3/gypsy elements, with subsequent vertical inheritance and limited recombination between env and pol domains. The paper also proposes an expanded definition of 'errantivirus' for env-carrying Ty3/gypsy elements outside Drosophila.

Strengths:

(1) Comprehensive Genomic Survey:

The breadth of the genome search across non-model metazoan phyla yields an impressive dataset covering evolutionary breadth, with clear documentation of search iterations and validation criteria for intact elements.

(2) Robust Phylogenetic Inference:

The use of maximum likelihood trees on both pol and env domains, with thorough congruence analysis, convincingly separates ancient from lineage-specific elements and demonstrates co-evolution of env and pol within clades.

(3) Structural Insights:

AlphaFold2-based predictions provide high-confidence structural evidence that both env types have retained fusion-competent architectures, supporting the hypothesis of preserved functional potential.

(4) Novelty and Scope:

The study challenges previous assumptions of insect-centric or recent env acquisition and makes a compelling case for a Pre-Cambrian origin, significantly advancing our understanding of animal retroelement diversity and evolution. THIS IS A MAJOR ADVANCE.

(5) Data Transparency:

I appreciate that all data, code, and predicted structures are made openly available, facilitating reproducibility and future comparative analyses.

Major Weaknesses

(1) Functional Evidence Gaps:

The work rests largely on sequence and structure prediction. No direct expression or experimental validation of envelope gene function or infectivity outside Drosophila is attempted, which would be valuable to corroborate the inferred roles of these glycoproteins in non-insect lineages. At least for some of these species, there are RNA-seq datasets that could be leveraged.

We added a sentence in the discussion, subsection “The survival mechanism of errantiviruses in the genome”, citing our recent work now published (PMID: 41922845), explaining that the defence mechanism against errantiviruses appears to be conserved in insects beyond *Drosophila*, indirectly suggesting that their biology dependent on the presence of env—may be more universal.

(2) Horizontal Transfer vs. Loss Hypotheses:

The discussion argues primarily for vertical inheritance, but the somewhat sporadic phylogenetic distributions and long-branch effects suggest that loss and possibly rare horizontal events may contribute more than acknowledged. Explicit quantitative tests for horizontal transfer, or reconciliation analyses, would strengthen this conclusion. It's also worth pointing out that, unlike retrotransposons that can be found in genomes, any potential related viral envelopes must, by definition, have a spottier distribution due to sampling. I don't think this challenges any of the conclusions, but it must be acknowledged as something that could affect the strength of this conclusion

We have added a targeted host-taxonomy concordance analysis for two well-sampled POL extended RT/connection subclades: an Annelida-associated clade from tree position A6 and a Lepidoptera-associated clade from tree position I1 (new Fig S5). Rather than attempting to infer exact numbers of duplication, loss and horizontal transfer events, which is difficult across highly expanded and unevenly sampled transposon families, we tested whether host-taxonomic labels were more clustered on the observed POL extended RT/connection topology than expected by chance. In the Annelida clade, highly supported small subclades showed strong host-family and host species concordance under host-label permutation tests. The Lepidoptera clade showed a more mixed pattern, but still contained several highly supported subclades enriched for related host groups at the superfamily or broader taxonomic level. These results do not exclude rare horizontal transfer, particularly between closely related hosts, but support the conclusion that the observed POL extended RT/connection trees retain significant host-taxonomic structure and are not consistent with frequent broad horizontal transfer between distantly related animal groups. We have added a paragraph in the Results section “Multiple intact elements of env-carrying Ty3/gypsy retrotransposons are found widespread across metazoan species” describing these observations, and also revised the Discussion to more explicitly acknowledge the possibilities of lineage-specific loss and the horizontal transfer.

(3) Limited Taxon Sampling for Certain Phyla:

Despite the impressive breadth, some ancient lineages (e.g., Porifera, Echinodermata) are negative, but the manuscript does not fully explore whether this reflects real biological absence, assembly quality, or insufficient sampling. A more systematic treatment of negative findings would clarify claims of ubiquity. However, I also believe this falls beyond the scope of this study.

In the revised manuscript, we have added a targeted analysis of two representative genomes from each phylum. Although we did not detect full-length GAG-POL-ENV elements in these genomes, we recovered multiple full-length, multicopy GAG-POL *Ty3/gypsy* elements from all four genomes, many of which were flanked by predicted LTR sequences and associated with putative tRNA primer-binding sites. This suggests that the apparent absence of env-carrying elements in these representative Porifera and Echinodermata genomes is unlikely to be due simply to poor assembly quality or a general inability to recover intact *Ty3/gypsy*-like retrotransposons. We have added these data as the new Supplementary table S3 and revised the Results section “Multiple intact elements of env-carrying Ty3/gypsy retrotransposons are found widespread across metazoan species” to clarify that absence in these phyla may reflect true biological absence, lineage-specific loss, or incomplete taxon sampling.

(4) Mechanistic Ambiguity:

The proposed model that env-containing elements exploit ovarian somatic niches is plausible but extrapolated from Drosophila data; for most taxa, actual tissue specificity, lifecycle, or host interaction mechanisms remain speculative and, to me, a bit unreasonable.

We stressed in the Discussion section “The survival mechanism of errantiviruses in the genome” that the mere presence of env gene does not imply the mechanism of transmission of retrotransposons.

Minor Weaknesses:

(1) Terminology and Nomenclature:

The paper introduces and then generalizes the term "errantivirus" to non-insect elements. While this is logical, it may confuse readers familiar with the established, Drosophila-centric definition if not more explicitly clarified throughout. I also worry about changes being made without any input from the ICTV nomenclature committee, which just went through a thorough reclassification. Nevertheless, change is expected, and calling them all errantiviruses is entirely reasonable.

We have revised the Results section and discussion where we introduced the term "errantivirus" to clarify that we use "errantivirus" operationally to refer to env-containing Ty3/gypsy retrotransposons identified in this study, rather than as a formal taxonomic proposal. We also now state explicitly that bona fide infectivity and amplification through the *Drosophila*-like ovarian somatic-cell route have not been experimentally established for most non-*Drosophila* elements. Our use of the term is therefore intended to distinguish env-containing Ty3/gypsy elements from related non-env containing Ty3/gypsy retrotransposons, while acknowledging that their biology outside *Drosophila* remains to be determined.

(2) Figures and Supplementary Data Navigation:

Some key phylogenies and domain alignments are found only in supplementary figures, occasionally hindering readability for non-expert audiences. Selected main-text inclusion of representative trees would benefit accessibility.

We agree that clearer navigation between the main text and supplementary figures would improve readability. Although we considered moving selected supplementary phylogenies and alignments into the main figures, the main figures are already data-dense and are intended to provide representative summaries across many host groups and ENV types. We therefore retained the detailed trees and alignments as supplementary figures, where they can be shown at readable scale, but revised the manuscript to improve navigation. Specifically, we added signposting sentences in the Results where supplementary figures are mentioned, expanded the relevant figure legends, and clarified how each supplementary tree or alignment supports the corresponding main-text conclusion.

(3) ORF Integrity Thresholds:

The cutoff choices for defining "intact" elements (e.g., numbers/placement of stop codons, length ranges) are reasonable but only lightly justified. More rationale or sensitivity analysis would improve confidence in the inclusion criteria. For example, how did changing these criteria change the number of intact elements?

We agree with the reviewer that the rationale for the ORF integrity thresholds should be stated more clearly. We have revised the Methods section "Identification of intact genomic copies of Ty3/gypsy errantiviruses" to clarify that the initial length, gap and stop-codon thresholds were deliberately permissive screening criteria, designed to avoid excluding divergent or non-canonical elements at the discovery stage. These initial filters were not used alone to define the final “intact” set. Candidate elements were subsequently subjected to multiple additional curation steps, including confirmation of Ty3/gypsy POL identity, recovery of full-length RT and Integrase domains within continuous ORFs, HHpred-based domain annotation of GAG, POL and ENV, and removal of elements with large domain truncations. Thus, the final set of intact elements is substantially more refined than would be implied by the initial stop codon or length thresholds alone.

A full sensitivity analysis varying each threshold across the entire iterative discovery and manual-curation pipeline would be difficult to interpret, because changing early permissive filters would alter the candidate pool that then undergoes downstream structural and phylogenetic validation. Instead, we have clarified in the Methods that the early thresholds were intended as inclusive prefilters, whereas final inclusion required intact domain architecture and phylogenetic/domain support.

(4) Minor Typos/Formatting:

The paper contains sporadic typographical errors and formatting glitches (e.g., misaligned figure labels, unrendered symbols) that should be addressed.

We now fixed these issues in the revised manuscript.

**Reviewer #2 (Public review):**

Summary:

The authors first surveyed metazoan genomes to identify homologs of Drosophila errantiviruses and classified them into two groups, "insect" and "ancient" elements, supporting the hypothesis of an early evolutionary origin for these retrotransposons. They subsequently identified two distinct types of envelope proteins, one resembling the glycoprotein F of paramyxoviruses and the other akin to the glycoprotein B of herpesviruses. Despite differences in their primary amino acid sequences, these proteins display notable structural similarity in their predicted domain architectures. The congruence between the phylogenies of the envelope and pol genes further supports the ancient origin of the envelope genes, challenging earlier hypotheses that proposed recent recombination events with baculoviruses. Additional analysis of the Pol "bridge region" corroborated the divergence among these elements, consistent with a pattern of limited cross-species recombination. Finally, by comparing these elements with non-envelope-containing Gypsy retrotransposons, the authors concluded that errantiviruses originated from multiple elements independently.

Strengths:

The conclusions of this study are based on a comprehensive collection of errantiviruses identified across a wide range of metazoan genomes. These findings are further supported by multiple lines of evidence, including phylogenetic congruence and the diverse evolutionary origins of envelope genes. AlphaFold2-assisted protein domain structure analyses also provided key insights into the characterization of these elements. Together, these results present a compelling case that errantiviruses arose independently through multiple evolutionary events, extending well beyond previous hypotheses.

Weaknesses:

It would be beneficial to emphasize in the Abstract the potential impact of this work by more clearly articulating the current knowledge gap in the field. While the second paragraph of the Introduction briefly touches on this point, highlighting the broader significance in the Abstract would better capture readers' interest. Additionally, some methodological choices would benefit from clearer justification and explanation. For instance, in Figure 6, the selection of the bridge region/RNase H domain is not explicitly explained, leaving the rationale for its choice unclear. As a minor point, some figure labels and texts are too small and difficult to read, and improving their legibility would enhance overall clarity.

We have revised the Abstract to more clearly state the knowledge gap addressed by this study: although env-containing Ty3/gypsy elements were known from *Drosophila* and sporadically reported in other animals, whether their association with env-like fusogen genes reflected recent, lineage-specific acquisitions or a much deeper evolutionary relationship remained unclear. We now highlight this broader significance in the Abstract and frame our results as evidence that env-containing Ty3/gypsy elements represent deeply diverged, genome-resident retroelements rather than a recent insect-specific phenomenon.

We have also revised the Results, Methods and Figure 6 legend to explain why the

RNase H-containing bridge region was analysed. Specifically, we now distinguish the Pol extended RT/connection region used for phylogenetic analysis from the RNase H-containing bridge region analysed structurally in Figure 6. We define the bridge region as the canonical RNase H domain together with the C-terminal region between RNase H and Integrase, and explain that this region was selected because RNase H-related and adjacent RNase H-like domains vary among LTR retroelement lineages. The bridge region architecture therefore provides an independent structural feature for comparing the “insect errantivirus” and “ancient errantivirus” groups.

Finally, we have revised the figures and figure legends to improve readability. In particular, we enlarged labels where possible, clarified figure annotations, corrected cross-references between main and supplementary figures, and added signposting sentences in the Results so that readers can more easily connect the main conclusions to the supporting supplementary trees and alignments.

**Reviewer #3 (Public review):**

Summary and Significance:

In this work, Cary and Hayashi address the important question of when, in evolution, certain mobile genetic elements (Ty3/gypsy-like non-LTR retrotransposons) associated with certain membrane fusion proteins (viral glycoprotein F or B-like proteins), which could allow these mobile genetic elements to be transferred between individual cells of a given host. It is debated in the literature whether the acquisition of membrane fusion proteins by non-LTR retrotransposons is a rather recent phenomenon that separately occurred in the ancestors of certain host species or whether the association with membrane fusion proteins is a much more ancient one, pre-dating the Cambrian explosion. Obviously, this question also touches upon the origin of the retroviruses, which can spread between individuals of a given host but seem restricted to vertebrates. Based on convincing data, Cary and Hayashi argue that an ancient association of non-LTR retrotransposons with membrane fusion proteins is most probable.

Strengths:

The authors take the smart approach to systematically retrieve apparently complete, intact, and recently functional Ty3/gypsy-like non-LTR retrotransposons that, next to their characteristic gag and pol genes, additionally carry sequences that are homologous to viral glycoprotein F (env-F) or viral glycoprotein B (env-B). They then construct and compare phylogenetic trees of the host species and individual encoded proteins and protein domains, where 3D-structure calculations and other features explain and corroborate the clustering within the phylogenetic trees. Congruence of phylogenetic trees and correlation of structural features is then taken as evidence for an infrequent recombination and a long-term co-evolution of the reverse transcriptase (encoded by the pol gene) and its respective putative membrane fusion gene (encoded by env-F or env-B). Importantly, the env-F and env-B containing retrotransposons do not form a monophyletic group among the Ty3/gypsy-like non-LTR retrotransposons, but are scattered throughout, supporting the idea of an originally ancient association followed by a random loss of env-F/env-B in individual branches of the tree (and rather rare re-associations via more recent recombinations).

Overall, this is valuable, stimulating, and important work of general and fundamental interest, but still also somewhat incompletely explored, imprecisely explained, and insufficiently put into context for a more general audience.

Weaknesses:

Some points that might be considered and clarified:

(1) Imprecise explanations, terms, and definitions:

It might help to add a 'definitions box' or similar to precisely explain how the authors decided to use certain terms in this manuscript, and then use these terms consistently and with precision.

(a) In particular, these are terms such as 'vertebrate retrovirus' vs 'retrovirus' vs 'endogenized retrovirus' vs 'endogenous retrovirus' vs 'non-LTR retrotransposon' and 'Ty3/gypsi-like retrotransposon' vs 'Ty3/gypsy retrotransposon' vs 'errantivirus'.

We agree with the reviewer. We inserted a paragraph at the end of the first Results section, explaining how we define endogenous retroviruses (ERVs), Ty3/gypsy retrotransposons and errantiviruses.

(b) The comment also applies to the term 'env' used for both 'env-F' and 'env-B', where often it remains unclear which of the two protein types the authors refer to. This is confusing, particularly in the methods, where the search for the respective homologs is described.

We revised the manuscript and now used F-type env/ENV and HSV/gB-type env/ENV throughout the text. We also modified the method section where we explained the tBlastn search to clarify which ENV proteins were used initially for the search and how we classified them in later analyses.

(c) Other examples are the use of the entire pol gene vs. pol-RT for the definition of the Ty3/gypsy clade and for the generation of phylogenetic trees (Methods and Figure S1), and the names for various portions of pol that appear without prior definition or explanation (e.g., 'pro' in Figure 1A, 'bridge' in Figure S1C, 'the chromodomain' in the text and Figure 7).

We revised the manuscript and explained ‘pro’, ‘bridge’ and ‘the chromodomain’ in the Results section or figure legends when they first appear. Please refer to other sections of the response for pol-RT definition.

(d) It is unclear from the main text which portions of pol were chosen to define pol-RT and why. The methods name the 'palm-and-fingers', 'thumb', and 'connections' domains to define RT. In the main text, the 'connection' domain is called 'tether' and is instead defined as part of the 'bridge' region following RT, which is not part of RT.

We agree that our previous terminology around Pol domains was imprecise and could confuse readers. We have revised the manuscript to distinguish the region used for phylogenetic analysis from the region analysed structurally in Figure 6. The phylogenetic analysis used an extended RT/connection region, comprising the RT polymerase core together with the downstream connection subdomain. This connection subdomain is treated as part of retroviral RT in structural studies, but corresponds to a partial RNase H-like fold and has been interpreted evolutionarily as a degenerated RNase H-like tether domain. It is therefore broader than the RT polymerase core alone, but it is not the complete canonical RNase H domain.

We now define the Figure 6 “bridge region” separately as the region spanning the canonical RNase H domain and the C-terminal region between RNase H and Integrase. Figure 6 shows that the invertebrate errantiviruses analysed retain an intact canonical RNase H domain immediately downstream of the extended RT/connection region, but differ in the additional downstream RNase H-like or mini-domain structures before Integrase. We have revised the Results, Methods and figure legends accordingly. We also acknowledge that a phylogeny based strictly on the RT polymerase core alone could differ in some local branch relationships, but the major conclusions are supported independently by the Integrase tree, host-taxonomic structure, ENV-type distribution, Pol bridge-region architecture and ENV structural features.

(2) Insufficient broader context:

(a) The introduction does not state what defines Ty3/gypsy non-LTR retrotransposons as compared to their closest relatives (Ty1/copia retrotransposons, BEL/pao retrotransposons, vertebrate retroviruses). This makes it difficult to judge the significance and generality of the findings.

(b) The various known compositions of Ty3/gypsi-like retrotransposons are not mentioned and explained in the introduction (open reading frames, (poly-)proteins and protein domains, and their variable arrangement, enzymatic activities, and putative functions), and the distribution of Ty3/gypsi-like retrotransposons among eukaryotes remains unclear. The introduction does not mention that Ty3/gypsi-like retrotransposons apparently are absent from vertebrates, and Figure 7 is not very clear about whether or not it includes sequences from plants ('Chromoviridae').

We agree that the Introduction needed more context on Ty3/gypsy retrotransposons. We have revised it to briefly state that LTR retrotransposons include several major lineages, including Ty1/copia, BEL/Pao, Ty3/gypsy and retrovirus-related elements, and that Ty3/gypsy elements are classified primarily by POL similarity and domain organisation. We also now explain that Ty3/gypsy retrotransposons typically encode GAG and POL proteins, with POL providing the enzymatic activities required for reverse transcription and integration, while noting that ORF arrangement and accessory domains can vary between lineages.

Please note that we stated that our screen did not identify intact env-containing Ty3/gypsy elements in vertebrate genomes that were homologous to the invertebrate errantiviruses analysed here. This is not to say that non-env-containing Ty3/gypsy elements are also absent in vertebrate genomes. Finally, we revised the Figure 7 legend to make clear that the comparison includes representative non-env-containing Ty3/gypsy elements from animals, fungi and plants, including chromovirus or chromovirus-related elements.

(c) The known association of Ty3/gypsi-like retrotransposons from different metazoan phyla with putative membrane fusion proteins (env-like) genes is mentioned in the introduction, but literature information, whether such associations also occur in the context of other retrotransposons (e.g., Ty1/ copia or BEL/pao), is not provided. The abstract is somewhat misleading in this respect. Finally, the different known types of env-like genes are not mentioned and explained as part of the introduction ('env-f', 'envB', 'retroviral env', others?)

We expanded the introduction to introduce literature information of known env-associated retroelements, including Ty1/copia and BEL/pao and explained which ENV types are known to be associated to these elements.

(d) Some key references and reviews might be added:

- Pelisson, A. et al. (1994) here (next to Song et al. (1994), for the identification of env in Ty3/gypsy)

- Boeke, J.D. et al. (1999) In Virus Taxonomy: ICTV VIIth report. (ed. F.A. Murphy),. Springer-Verlag, New York. (cited by Malik et al. (2000) - for the definition and first use of the term 'errantivirus')

- Eickbush, T.H. and Jamburuthugoda, V.K. (2008) here (on the classification of retrotransposons and their env-like genes)

- Hayward, A. (2017) here (on scenarios of env acquisition)

Thank you. We included these references in the introduction.

(3) Incomplete analysis:

(a) Mobile genetic elements are sometimes difficult to assemble correctly from shortread sequencing data. Did the authors confirm some of their newly identified elements by e.g., PCR analysis or re-identification in long-read sequencing data?

Most newly identified elements are found in contigs/chromosomes that are longer than 100kb. The information of the contig/chromosome size, in which the representative copy of the identified elements are found, can be found in the column “CONTIG_SIZE” in supplementary table S1.

(b) The authors mention somewhat on the side that there are Ty3/gypsy elements with a different arrangement (gag-env-pol instead of gag-pol-env). Why was this important feature apparently not used and correlated in the analysis? How does it map on the RT phylogenetic tree? Which type of env is found with either arrangement? Is there evidence for a loss of env also in the case of gag-env-pol elements?

We agree that the non-canonical GAG-ENV-POL arrangement is an important feature that was insufficiently integrated into the analysis. We have revised the Results and figure annotations to make this clearer. Specifically, we now indicate GAG-ENV-POL elements in the POL tree in Fig S4 and in the HSV/gB-type ENV alignment/architecture figure S8. These elements are found in Nematoda, Bryozoa and Platyhelminthes and all carry HSV/gB-type ENV. They do not form a single monophyletic group in the Pol tree, but instead occur in distinct host-associated clades. They also show different HSV/gBtype cysteine-bridge architectures. Thus, the GAG-ENV-POL arrangement is unlikely to represent a single recent rearrangement event shared by all such elements; rather, it appears to be associated with several deeply diverged HSV/gB-type errantivirus lineages.

We have not inferred specific env-loss events for GAG-ENV-POL elements, because doing so would require a separate analysis of related non-env-containing elements.

(c) Sankey plots are insufficiently explained. How would inconsistencies between trees (recombinations) show up here? Why is there no Sankey plot for the analysis of env-B in Figure 5?

We agree that the Sankey plot was insufficiently explained. We have revised the Figure 4 legend to clarify that the Sankey plot was used as a qualitative visual summary of global congruence between the Pol extended RT/connection phylogeny and the F-type ENV ectodomain phylogeny. We now state that ribbon crossing alone should not be interpreted as recombination, because tree drawings can be rotated without changing topology and the relative order of clades in the two displayed trees may differ. Instead, the relevant signal is whether Pol-defined clades map mostly to corresponding F-type ENV-defined clades. Strong discordance, potentially reflecting recombination, env exchange or poor phylogenetic resolution, would be expected to appear as extensive splitting or many-to-many connections between Pol and ENV clades.

We did not include an equivalent Sankey plot for HSV/gB-type ENV in Figure 5 because we did not construct a global HSV/gB-type ENV phylogeny comparable to the F-type ENV ectodomain tree. Instead, HSV/gB-type ENV proteins were analysed by predicted structural organisation and cysteine-bridge architecture, which are shown in Figure 5 and Supplementary Figure S8.

(d) Why are there no trees generated for env-F and env-B like proteins, including closely related homologous sequences that do NOT come from Ty3/gypsy retrotransposons (e.g., from the eukaryotic hosts, from other types of retrotransposons (Ty1/copia or BEL/pao), from viruses such as Herpesvirus and Baculovirus)? It would be informative whether the sequences from Ty3/gypsy cluster together in this case.

We agree that comparison with homologous fusogens outside Ty3/gypsy retrotransposons is informative. We have therefore added an expanded F-type ENV ectodomain phylogeny that includes representative viral and retroelement-associated F-like proteins, including baculovirus F proteins, paramyxovirus and pneumovirus F proteins, and the BEL/Pao-associated *Drosophila* Roo F-like protein. In this expanded tree, the added viral sequences formed family-level clades within the broader F-type ENV diversity. Errantivirus F-type ENV proteins did not cluster as a shallow Ty3/gypsyspecific group or as a recent derivative of a single sampled viral family; instead, they spanned a level of diversity comparable to that separating major viral F-protein groups.

For HSV/gB-type ENV proteins, we did not generate an equivalent global phylogeny because the primary sequences and domain organisations of viral class III fusogens and errantivirus HSV/gB-type ENV proteins were too divergent for reliable full ecto domain multiple-sequence alignment. Instead, we added a structural comparison with representative viral and retroelement-associated class III fusogens, including herpesvirus gB, rhabdovirus G, orthomyxovirus GP75/GP64-like proteins, baculovirus GP64 and BEL/Pao-associated gB-like proteins. This analysis showed that viral class III fusogens often retained family-specific cysteine-bridge architectures despite low primary-sequence identity. Errantivirus HSV/gB-type ENV groups showed a comparable pattern, retaining lineage-specific cysteine-bridge architectures despite extensive sequence divergence. We have revised the Results, Methods and supplementary figure legends to clarify these analyses and to distinguish the phylogenetic analysis of F-type ENV from the structural comparison of HSV/gB-type ENV.

(e) Did the authors identify any other env-like ORFs (apart from env-F and env-B) among Ty3/gypsy retrotransposons? Did they identify other, non-env-like ORFs that might help in the analysis? It is not quite clear from the methods if the searches for env-F and envB - containing Ty3/gypsy elements were done separately and consecutively or somehow combined (the authors generally use 'env', and it is not clear which type of protein this refers to).

We agree that this was not sufficiently clear. We have revised the Methods to clarify that the search was designed to identify Ty3/gypsy elements carrying ORFs structurally resembling known envelope/fusogen proteins. In the iterative tBLASTn searches, bait sequences representing both F-type ENV and HSV/gB-type ENV were included together in each round, rather than being searched as two entirely separate pipelines. Candidate elements were then annotated and classified by ORF structure, HHpred/domain similarity and structural prediction.

Among intact Ty3/gypsy candidates recovered by this strategy, we identified two recurrent classes of env-like ORFs: F-type env and HSV/gB-type env. We did not identify an additional recurrent class of env-like ORF among the intact Ty3/gypsy elements analysed here. We also did not identify other recurrent non-env accessory ORFs that were informative for the phylogenetic analyses beyond the GAG, POL and ENV features described in the manuscript.

(f) Why was the gag protein apparently not used to support the analysis? Are there different, unrelated types of gag among non-LTR retrotransposons? Does gag follow or break the pattern of co-evolution between RT and env-F/env-B?

We agree that the role of GAG in the analysis should be clarified. GAG ORFs were used during element annotation to identify intact GAG-POL-ENV or GAG-ENV-POL retrotransposon architectures, but we did not use GAG as a major phylogenetic marker because GAG proteins are less conserved and less reliably alignable across deeply diverged Ty3/gypsy elements than the enzymatic POL domains. Our central question was the acquisition and long-term retention of env-like ORFs by POL-defined Ty3/gypsy retrotransposons. We have revised the Methods to clarify that GAG was used for structural annotation and intactness assessment, whereas phylogenetic analyses were based on the Pol extended RT/connection region and Integrase domain.

(g) Data availability. The link given in the paper does not seem to work (here). It would be useful for the community to have the sequences of the newly identified Ty3/gypsy retrotransposons listed readily available (not just genome coordinates as in table S1), together with the respective annotations of ORFs and features.

The GitHub repository that contains suggested data is made public. Please check the link again.

**Recommendations for the authors:**

**Reviewer #1 (Recommendations for the authors):**

Additional Analyses That Could Strengthen Claims (but I concede might be well beyond the scope of this study):

(1) Reconciliation and Gene Tree-Species Tree Analysis: Implementing explicit genetree-species-tree reconciliation (e.g., using Notung or ALE) could formally test the frequency of horizontal transfers vs. vertical inheritance in errantivirus evolution.

We performed targeted host-taxonomy concordance analyses in representative well-sampled clades to address this question, as described in the revised manuscript.

(2) Functional Validation in Non-Insect Hosts: RNA-seq or proteomics from representative non-insect hosts could reveal whether env genes are expressed, and-if possible-experimental assays for envelope function would move beyond computational inference. This is the one thing that might be done in a revision.

We agree that direct functional validation in non-*Drosophila* species would substantially strengthen the conclusions. Such experiments are beyond the scope of the present revision. However, we now cite our recent work (PMID: 41922845) showing conserved piRNA-mediated defence against errantiviruses across insect orders, providing indirect evidence that these elements remain biologically active outside *Drosophila*.

(3) LTR Age Dating: Estimating insertion ages using LTR divergence across clades would contextualize the timing of expansion events and help test hypotheses about ancient vs. recent proliferation.

We agree that LTR divergence-based insertion dating would be informative for estimating the timing of recent expansion events. However, our main evolutionary conclusions concern the deeper history of env acquisition and long-term retention across metazoan lineages, rather than the precise insertion age of individual genomic copies. Because the dataset includes elements from highly divergent genomes with variable assembly quality and many multicopy families, systematic LTR dating across all clades would require additional curation and is beyond the scope of the present revision.

(4) Comparative Host Defense Analysis: Surveying host antiviral or transposon defense systems (e.g., piRNA, APOBEC) in lineages rich in errantiviruses could test for signatures of recurrent molecular arms races.

We agree that comparative analysis of host defence pathways, including piRNA and antiviral systems, is an important future direction. However, a systematic survey of host-defence evolution across all errantivirus-rich lineages is beyond the scope of the present revision.

**Reviewer #2 (Recommendations for the authors):**

Apart from my comments in the Public Review, I have a few additional minor recommendations:

(1) The authors frequently use the term "active" to describe complete retrotransposons. However, in transposon biology, "active" implies recent or ongoing transpositional activity and should therefore be used with caution. Terms such as "complete" or "fulllength" would be more appropriate in this context.

We agree that intact ORF structure should not be over-interpreted as evidence of recent mobilisation. We have therefore revised the manuscript to distinguish element intactness from evidence of recent expansion. Specifically, we now use “intact” or “full-length” to describe element structure. We also clarify that the presence of multiple highly similar copies in the same genome, defined as >98% nucleotide identity across >98% of the three ORFs, is evidence consistent with recent or ongoing genomic expansion, but not definitive proof of current transposition. These changes have been made in the Abstract, Results, Methods and Discussion.

(2) In the third paragraph of the Results, the authors conclude that many identified errantiviruses were mobilized recently. However, since the search strategy specifically targets complete and uninterrupted elements, this may introduce a bias toward younger elements, making the conclusion somewhat circular.

We agree and have revised the wording to avoid over-interpreting completeness as evidence of recent mobilisation. We now distinguish between intact/full-length element structure, which was part of our search strategy, and independent evidence for recent or ongoing mobilisation, such as the presence of multiple highly similar copies. We have therefore softened statements that previously implied that intact ORFs alone demonstrate recent activity.

(3) The first paragraph of the Introduction requires appropriate references.

We have now added references to enhance the readability of the first paragraph of the introduction.

(4) It would benefit readers if the retrotranspositional process were briefly explained, including a description of what the tRNA primer binding site (PBS) is and its role in retrotransposition.

In the same part of the introduction, we now describe the role of the tRNA primer binding site in retrotransposition.

**Reviewer #3 (Recommendations for the authors):**

Suggestions and requests for clarification currently are part of the public review, as they also point the reader to critical open questions if the authors decide not to amend their version of the manuscript.
